# Electromyographic Patterns of Masticatory Muscles in Relation to Active Myofascial Trigger Points of the Upper Trapezius and Temporomandibular Disorders

**DOI:** 10.3390/diagnostics11040580

**Published:** 2021-03-24

**Authors:** Grzegorz Zieliński, Aleksandra Byś, Jacek Szkutnik, Piotr Majcher, Michał Ginszt

**Affiliations:** 1 Department of Rehabilitation and Physiotherapy, Medical University of Lublin, 20-093 Lublin, Poland; grzegorz.zielinski@umlub.pl (G.Z.); aleksandra.bys@umlub.pl (A.B.); piotr.majcher@umlub.pl (P.M.); 2 Department of Functional Masticatory Disorders, Medical University of Lublin, 20-093 Lublin, Poland; zakladzaburzen@umlub.pl

**Keywords:** electromyography, temporalis anterior, masseter muscle, myofascial pain, myofascial trigger points, trapezius

## Abstract

The presented study aimed to analyze and compare the electromyographic patterns of masticatory muscles in subjects with active myofascial trigger points (MTrPs) within upper trapezius, patients with temporomandibular disorders (TMDs) and healthy adults. Based on the diagnostic criteria of MTrPs according to Travell & Simons and the Research Diagnostic Criteria for Temporomandibular Disorders, 167 people were qualified for the study. Subjects were divided into 3 groups: with active MTrPs in the upper trapezius, with diagnosed temporomandibular disorders (TMDs) and healthy adults. Measurements of the bioelectric activity of the temporalis anterior (TA) and masseter muscle (MM) were carried out using the BioEMG III ™. Based on statistical analysis, significantly lower values of TA resting activity were observed among controls in comparison to MTrPs (1.49 μV vs. 2.81 μV, *p* = 0.00) and TMDs (1.49 μV vs. 2.97 μV, *p* = 0.01). The POC index values at rest differed significantly between MTrPs and TMDs (86.61% vs. 105%, *p* = 0.04). Controls presented different electromyographic patterns within AcI in comparison to both MTrPs (4.90 vs. −15.51, *p* = 0.00) and TMDs (4.90 vs. −16.49, *p* = 0.00). During clenching, the difference between MTrPs and TMDs was observed within MVC TA (91.82% vs. 116.98%, *p* = 0.02). TMDs showed differences within AcI in comparison to both MTrPs group (−42.52 vs. 20.42, *p* = 0.01) and controls (−42.52 vs. 3.07, *p* = 0.00). During maximum mouth opening, differences between MTrPs and TMDs were observed within the bioelectric activity of masseter muscle (16.45 μV vs. 10.73 μV, *p* = 0.01), AsI MM (0.67 vs. 11.12, *p* = 0.04) and AcI (13.04 vs. −3.89, *p* = 0.01). Both the presence of MTrPs in the upper trapezius and TMDs are related to changes in electromyographic patterns of masticatory muscles.

## 1. Introduction

Myofascial trigger points (MTrPs) are defined as hyperactive points located in the tense area of the skeletal muscle or its fascia. MTrPs are associated with the development of myofascial pain syndrome (MPS), causing local or referred pain [1]. The compression stimulation of MTrPs may induce a local pain sensation or a referred pain response [2]. The development of MTrPs may be caused by the accumulation of microtraumas within the muscle or its direct injury [1,3]. Muscle overload and consequently the formation of MTrPs, is the result of prolonged or repeated low-amplitude muscle contractions, eccentric contractions and maximal or submaximal muscle contractions [3,4]. Moreover, MTrPs can arise as a result of nutrient deficiencies, hormonal disorders or muscle imbalances [4], fatigue and even viral infections [2,5]. The pathology mentioned above may be related to tissue hypoxia processes in the MTrPs environment [6] when the concentration of inflammatory mediators increases near MTrPs [7]. The above factors may lead to increased nociceptor activity, which results in increased pain response [8]. It is estimated that the prevalence of MPS in clinical populations varies widely, ranging from 9% to 85% [9].

The temporomandibular joint (TMJ) is a bilateral joint composed of the temporal bone′s articular surface and the head of the mandible [10]. TMJ is separated into two synovial joint cavities by an articular disc, allowing a smooth articulation between the mandibular condyle and the articular eminence. Moreover, the TMJ disc increases the contact area between opposing articulating surfaces, distributing lower stresses to a larger surface area in the joint [11]. The anterior portion of the TMJ disc is attached to the joint capsule, articular eminence, anterior condyle and the lateral pterygoid′s upper area. The posterior portion attaches superiorly to the temporal bone and inferiorly to the posterior condyle. Several ligaments, TMJ disc, articular capsule and masticatory muscles stabilize the TMJ and manage the TMJ forces [12]. Both TMJ dysfunction and abnormalities within masticatory muscles may lead to Temporomandibular Disorders (TMDs). TMDs affect the TMJ, masticatory muscles and/or surrounding tissues and are mainly characterized by pain, acoustic symptoms and limited, incorrect or parafunctional muscle activity [13]. The most common conditions comprising TMDs are myofascial pain, disc displacements and TMJ arthritides [13]. In addition, MPS associated with the presence of MTrPs accounts for approximately 45% of all reported cases of TMDs [14]. Moreover, TMDs significantly reduced life quality and are recognized by the World Health Organization as the third most common dental disease [15,16]. The American National Institute of Dental and Craniofacial Research estimates that TMDs affect 5 to 12% of the population, more often women than men [17]. However, TMDs′ etiology is multifactorial and still unclear, with some suggesting that due to their association with other somatic syndromes, TMDs may be part of the same phenomenon [18].

The phenomenon of referred pain is the subject of discussion concerning the stomatognathic system disorders. However, the mechanisms causing this phenomenon have not been clearly explained [19]. Trigger points in the upper trapezius have been associated with tension-type headache episodes [20]. Therefore, through the mechanism of referred pain, MTrPs in the upper trapezius may be responsible for developing pain within the masticatory muscles. The association between TMDs and disorders within cervical spine muscles remaining unclear and there are just several studies confirming the relationship between MTrPs in the cervical muscles and TMDs [21]. Previous reports indicate the coexistence of MTrPs in the neck muscles in patients with TMDs [14,19,22]. However, according to the authors′ knowledge, there is a lack of studies that have analyzed the relationship between active MTrPs of the trapezius muscle and the masticatory muscle activity. Thus, the presented study aimed to determine, analyze and compare electromyographic patterns of masticatory muscles in relation to active MTrPs of the upper trapezius and TMDs. Based on the above-mentioned interactions between MTrPs in cervical spine muscles and the occurrence of TMDs, we hypothesize that MTrPs within the upper trapezius significantly influence the activity of the masticatory muscles. We also assume that the electromyographic patterns of masticatory muscles in individuals with MTrPs within trapezius and TMDs patients will differ from healthy individuals.

## 2. Materials and Methods

The study was carried out in accordance with the recommendations of the Helsinki Declaration and with the consent of the Bioethical Commission of the Medical University of Lublin (approval number KE-0254/346/2016, date of approval 23.11.2016). All participants were informed about the aim of the study and have given written consent for the research.

The inclusion criteria used in the presented study were: age range 18–35 years, good or very good general health status according to the RDC/TMD questionnaire, the presence of active MTrPs in the upper trapezius and absence of any type of TMDs (MTrPs group), presence of pain-related TMDs based on the Research Diagnostic Criteria for Temporomandibular Disorders RDC/TMD [23] without MTrPs in the upper trapezius (TMDs group) and absence of TMDs and active and/or latent MTrPs in the head and neck muscles (control group).

The diagnostic of pain-related TMDs was performed by an experienced dentist with a specialization in dental prosthetics. The TMDs group included only patients with masticatory muscle disorders diagnosed with myalgia—myofascial pain. Patients with temporomandibular joint disorders (e.g., joint pain, disc disorders, joint diseases), other masticatory muscle dysfunctions (e.g., contracture, tendonitis, myositis, spasm, hypertrophy), fibromyalgia, headaches attributed to TMD and coronoid hyperplasia were excluded from the presented study [24].

The presence of active MTrPs within the upper trapezius was established by the following diagnostic criteria according to Travell and Simons [2].

- the presence of a taut band within the above-mentioned muscle;
- presence of a tender nodule within the taut band;
- recognizing pain as previously felt under pressure from a taut band;
- the appearance of radiating pain under pressure.

The following exclusion criteria were used: skin diseases in the head and neck area, neurological disorders in the head and neck area, neoplastic diseases (regardless of type and location), head and neck injuries within the last 6 months before the examination, surgical treatment in the area of head and neck in the last 6 months before the examination, class II and III according to Angle′s classification, class I malocclusions patients, open bite, having an orthodontic appliance, lack of four support zones in dental arches, lack of more than four teeth within both dental arches and possession of dental prostheses (regardless of type). After applying the above criteria, 167 people (age 26 ± 8 years) were divided into three groups: 60 in the MTrPs group, 47 in the TMDs group and 60 controls (Table 1).

**Figure 1:**
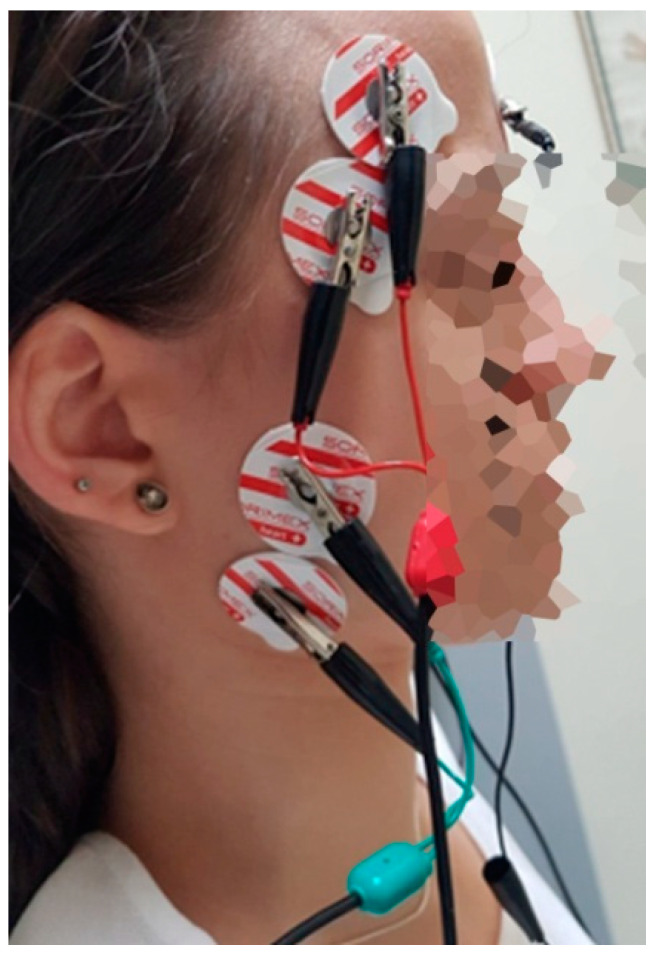
Electrodes placement during the electromyographic examination.

In the next stage, an electromyographic examination was carried out, which was always performed in the morning hours (9 am–11 am) to reduce the impact of the daily bioelectric variability of muscles on the results. The subjects sat on the dental chair, the head rested on the headrest and the torso was perpendicular to the ground. The lower limbs were straight, relaxed and parallel. Before electrode placement, the skin was cleansed with a 90% ethyl alcohol solution to reduce electrode–skin impedance. Ag/AgCl electrodes (SORIMEX, Poland) with a diameter of 30 mm and a conductive surface of 16 mm were used. The placement of the surface electrodes was performed following the Surface Electromyography for Non-invasive Assessment of Muscles (SENIAM) project [25]. The surface electrodes were placed on o the temporalis anterior (TA) and the superficial part of the masseter muscle (MM) in accordance with the course of the muscle fibers, according to the placement technique described by Ferrario et al. [26]. The reference electrode was placed on the forehead (Figure 1). An 8-channel BioEMG III^TM^ surface electromyography apparatus with BioPak Measurement System (BioResearch Associates, Inc. Milwaukee, WI, USA) was used for the study.

The activity of the masticatory muscles (TA, MM) was recorded in the following protocol: during resting mandibular position (10 s), during maximum voluntary clenching (three clenches of 3 s, each with a 2-s break), during maximum voluntary clenching on dental cotton rollers (three clenches of 3 s, with a 2-s break) and during maximum mouth opening (three abductions of 3 s, with a 2-s rest between) [27,28].

The electromyographic signals were amplified and purified from 99% of the noise scale on a linear scale using the BioPak digital NoiseBuster filter.

Based on the bioelectric data obtained, the following indices were calculated according to standardized protocols:

- MCV (maximum voluntary contraction) based on the formula [28]:
  MCV = [voluntary teeth clenching/voluntary teeth clenching on cotton rollers] × 100%
- POC (percentage overlapping coefficient) based on the formula [29]:
  POC = [(MM_right_ + TA_right_)/(MM_left_ + TA_left_)] × 100%
- AsI (asymmetry index) based on the formula [30]
  ASI =  [(RMS_right_ − RMS_left_)/(RMS_right_  +  RMS_left_)] × 100
- AcI (activity index) based on the formula [30]:
  ACI  =  [(RMS_masseter_ − RMS_temporal_)/(RMS_masseter_  +  RMS_temporal_)] × 100
- TC (torque) based on the formula [31]:
  TC = [(TA_right_ + MM_left_) − (TA_left_ + MM_right_)] × 100%

The checklist developed by the Strengthening the Reporting of Observational Studies in Epidemiology (STROBE) initiative was used to assess the methodological quality of the presented study [32]. IBM SPSS Statistics 21 software was used for statistical analysis. First, the normality of the distribution of variables was verified using the Shapiro-Wilk test and the Kolmogorov–Smirnov test (with Lillierfors correction). All the distributions were abnormal; therefore, the Kruskal—Wallis test was used. The significance level was set at 0.05. When there were significant differences between the analyzed groups, the post-hoc test was applied (Dunn′s Test).

## 3. Results

### 3.1. General Characteristics of Participants

There were no significant differences in the number of participants and gender between study groups and controls. Post-hoc analysis showed considerable age differences between the TMDs and the rest of the groups (MTrPs group and controls) (Table 1).

There were significant differences in the mandibular range of motion (ROM) between TMDs group vs. controls and TMDs vs. MTrPs. TMDs presented a decrease within the maximum comfortable pain-free opening (MCO), maximum mouth opening (MMO) and protrusion compared to other groups. Moreover, statistical analysis showed differences in the right lateral excursion (RLE) between the TMDs and controls. The mean mandibular ROM values were similar between MTrPs and controls (Table 2).

### 3.2. Electromyographic Analysis of Resting Masticatory Muscle Activity

Based on statistical analysis, significantly lower values of TA resting activity were observed among controls in comparison to MTrPs (Controls: 1.49 μV vs. MTrPs: 2.81 μV; *p* = 0.00) and TMDs (Controls: 1.49 μV vs. TMDs: 2.97 μV; *p* = 0.01), as presented in Table 2. The values of POC index at rest differed significantly between MTrPs and TMDs (MTrPs: 86.61% vs. TMDs: 105%; *p* = 0.04). Significant differences in electromyographic patterns between MTrPs and the other groups were also observed for the AsI TA (MTrPs: −14.72 vs. TMDs: −1.48 and Controls: −4.48; *p* = 0.00 and *p* = 0.01, respectively) and TC (MTrPs: −90.43% vs. TMDs: 0.28% and Controls: −3.67%; *p* = 0.02 and *p* = 0.03, respectively). Controls presented different electromyographic patterns within AcI in comparison to both MTrPs (Controls: 4.90 vs. MTrPs: −15.51; *p* = 0.00) and TMDs (Controls: 4.90 vs. TMDs: −16.49; *p* = 0.00) (Table 3).

### 3.3. Electromyographic Analysis of Masticatory Muscle Activity during Clenching

During clenching, difference between MTrPs and TMDs was observed within bioelectric activity of masseter muscle (MTrPs: 120.43 μV vs. TMDs: 68.30 μV; *p* = 0.00) and MVC TA (MTrPs: 91.82% vs. TMDs: 116.98%; *p* = 0.02). Moreover, differences between TMDs and controls were obserwed within bioelectric activity of TA (TMDs: 89.56 μV vs. Controls: 118.37 μV; *p* = 0.03) and MM (TMDs: 68.3 μV vs. Controls: 133.63 μV; *p* = 0.00). In addition, TMDs showed differences within AcI in comparison to both MTrPs group (TMDs: −42.52 vs. MTrPs: 20.42; *p* = 0.01) and controls (TMDs: −42.52 vs. Controls: 3.07; *p* = 0.00) (Table 4).

### 3.4. Electromyographic Analysis of Masticatory Muscle Activity during Maximum Mouth Opening

During maximum mouth opening, differences between MTrPs and TMDs were observed within the bioelectric activity of masseter muscle (MTrPs: 16.45 μV vs. TMDs: 10.73 μV; *p* = 0.01), AsI MM (MTrPs: 0.67 vs. TMDs: 11.12; *p* = 0.04), AcI R (MTrPs: 14.35 vs. TMDs: −0.23; *p* = 0.03), AcI L (MTrPs: 11.32 vs. TMDs: −11.06; *p* = 0.00) and AcI (MTrPs: 13.04 vs. −3.89; *p* = 0.01). Moreover, TMDs showed differences within AcI L in comparison to controls (TMDs: −11.06 vs. Controls: 7.65; *p* = 0.02) (Table 5). In terms of other indices, the differences between the studied groups did not reach the assumed significance level (Table 3, Table 4 and Table 5).

## 4. Discussion

The referred pain induced from active MTrPs in the neck muscles shared a similar pain pattern as spontaneous TMDs [19]. Thus, MTrPs in the upper trapezius may be responsible for the development of pain within the masticatory muscles. However, the association between TMDs and disorders within trapezius remaining unclear. Thus, the presented study aimed to determine, analyze and compare electromyographic patterns of masticatory muscles in relation to active MTrPs of the upper trapezius and TMDs. To our knowledge, this is the first study to evaluate electromyographic patterns of masticatory muscles in relation to active myofascial trigger points of the upper trapezius and temporomandibular disorders. We hypothesized that MTrPs within the upper trapezius significantly influence the activity of the masticatory muscles. We also assumed that the electromyographic patterns of masticatory muscles in the group with MTrPs within trapezius and TMDs patients would be different from healthy individuals.

During the electromyographic examination, significantly higher values of resting activity within temporalis anterior were observed among both MTrPs and TMDs patients in comparison to healthy individuals. The above-mentioned association was not observed within masseter muscle. Moreover, the differences within the distribution of resting muscle activity between the temporalis anterior and the masseter muscle significantly influenced activity index values in both studied groups. Both MTrPs and TMDs patients showed negative (−). AcI values, compared to healthy individuals whose AcI values were slightly positive (+). Negative values of AcI among MTrPs and TMDs indicate the predominance of the temporalis anterior during rest, in contrast to healthy controls with slight positive AcI values (masseter muscle advantage). However, the electromyographic patterns of teeth clenching differ significantly between MTrPs and TMDs patients regarding the activity index. The positive values of AcI during clenching showed the predominance of masseter muscle activity among individuals with active MTrPs within trapezius, unlike TMDs patients with negative values of AcI, indicating the predominance of the temporalis anterior during clenching tasks. In addition, the MVC index within the TA was significantly lower in MTrPs patients than in TMDs and healthy participants. Different electromyographic patterns between TMDs and MTrPs were also observed during maximum mouth opening in terms of bioelectric activity of the masseter muscle, as well as AsI MM and AcI indices.

The above changes in the masticatory muscle activity seem to be related to the integrated pain adaptation model, which assumes a new muscle activation strategy to maintain homeostasis [33]. The presented model postulates that the key factor in maintaining homeostasis may be the need to minimize the generation of further pain at rest or during movement. Thus, changes in the electromyographic patterns of masticatory muscles may be associated with the presence of pain due to active MTrPs. Previous studies indicate the association between the active MTrPs within masticatory muscles, increased muscle activity during rest and a decrease in sEMG values during teeth clenching [34,35,36]. The above-mentioned association may be linked with TA resting activity obtained in our work, both in TMDs and MTrPs groups, showing a similar resting activity pattern in both groups of patients.Note, however, that the AcI values differed significantly between MTrPs and TMDs during clenching tasks. The predominance of TA muscle activity in TMDs patients could be caused by reducing contraction patterns within the MM, which seems to be confirmed in the Mapelli et al. study [37]. However, the MTrPs group presented an entirely different electromyographic pattern with decreased temporalis anterior activity, both during teeth clenching and maximum mouth opening. We suspect that this altered pattern may be related to the occurrence of active MTrPs in the trapezius muscle, which, as a result of a referred pain mechanism, alters TA activity. Our suppositions seem to be in line with the referred pain patterns presented by Travell and Simons, in which the temporal area is one of the most commonly painful regions raised from MTrPs located in the upper trapezius [38]. As, based on our data, we cannot directly confirm this mechanism, we should treat this as a supposition and future studies should test if this mechanism is true.

Our hypothesis that MTrPs within the upper trapezius significantly influence the masticatory muscle activity seems to be confirmed in the presented research. This notion is in line with the results of previous findings showing the relationship between MTrPs in the upper trapezius and tension-type headache episodes [20,39,40,41,42,43]. In addition, the presence of bilateral pain hypersensitivity in the trigeminal region in patients with idiopathic neck pain was observed in La Touche et al. study, which suggests a sensitization process of the trigeminocervical nucleus [44]. Moreover, a study conducted by De-la-Llave-Rincon et al. suggests that chronic pain in the cervical region influences the formation of latent trigger points in the masticatory muscles [45]. The relationship between the masticatory muscles and the pain within the cervical area seems to be confirmed by Testa et al. [46]. In the above-mentioned study, patients with chronic pain in the cervical spine region presented the altered distribution of the electromyographic patterns within masticatory muscles during clenching. The authors also suggest that changes in the activity of the masticatory muscles observed in patients with cervical spine pain patterns may affect the development of TMDs.

Our assumption that the electromyographic patterns of masticatory muscles in the group with MTrPs within trapezius and in TMDs patients will be different from healthy individuals seems to be justified by obtained results. However, we cannot clearly explain the significant differences observed between MTrPs and TMD within the electromyographic patterns, which requires further research.The presented study has several limitations. Firstly, the diagnostics criteria for TMDs were changed to DC/TMD in 2014. However, there is no validated Polish version of the DC/TMD so far. Therefore, we used the clinical examination based on the Axis-I protocol of the RDC/TM. Moreover, the Axis I section of the RDC/TMD form is widely used in the current literature in high-impact journals [47,48,49,50]. Secondly, the study sample consists of young adults aged 18 to 35. Thus, future research should include a population with an expanded age range.

## 5. Conclusions

Both the presence of MTrPs in the upper trapezius and TMDs are related to changes in electromyographic patterns of masticatory muscles. Future research is needed to explain the above differences and underlying mechanisms.

## Figures and Tables

**Table 1:** General characteristics of participants.

	MTrPs Group	TMDs Group	Control Group	*p* Value MTrPs vs. TMDs	*p* Value MTrPs vs. Controls	*p* Value TMDs vs. Controls
N	60	47	60	1.00	1.00	1.00
Female	42	33	42	1.00	1.00	1.00
Male	18	14	18	1.00	1.00	1.00
Age	23 ± 3	33 ± 1	23 ± 3	0.00 *	1.00	0.00 *

* Significant difference.

**Table 2:** Comparison of the mean values (± SD) of mandibular range of motion during maximum comfortable pain-free opening (MCO), maximum mouth opening (MMO), right lateral excursion (RLE), left lateral excursion (LLE) and protrusion between groups.

	MTrPs Group	TMDs Group	Control Group	*p* Value MTrPs vs. TMDs	*p* Value MTrPs vs. Controls	*p* Value TMDs vs. Controls
MCO (mm)	50.13 ± 7.38	35.13 ± 13.08	51.13 ± 6.12	0.00 *	1.00	0.00 *
MMO (mm)	51.22 ± 7.49	42.51 ± 11.05	51.57 ± 6.10	0.00 *	1.00	0.00 *
RLE (mm)	9.00 ± 2.54	7.62 ± 3.52	9.17 ± 2.82	0.18	1.00	0.03 *
LLE (mm)	9.50 ± 2.61	8.34 ± 3.60	9.27 ± 3.21	0.20	1.00	0.43
Protrusion (mm)	7.48 ± 2.49	5.15 ± 2.59	7.60 ± 2.61	0.00 *	1.00	0.00 *

* Significant difference.

**Table 3:** Comparison of the mean values (± SD) of resting bioelectric activity of temporalis anterior (TA), masseter muscle (MM) and electromyographic indices between groups.

		MTrPs Group	TMDs Group	Control Group	*p* Value MTrPs vs. TMDs	*p* Value MTrPs vs. Controls	*p* Value TMDs vs. Controls
Rest	TA (µV)	2.81 ± 1.23	2.97 ± 2.42	1.49 ± 0.51	0.14	0.00 *	0.00 *
	MM (µV)	2.05 ± 1.03	1.81 ± 1.25	1.72 ± 0.83	0.10	0.32	1.00
	POC (%)	86.61 ± 26.78	105.00 ± 40.60	95.92 ± 21.88	0.04 *	0.07	1.00
	AsI TA	−14.72 ± 20.60	−1.48 ± 21.31	−4.48 ± 12.13	0.00 *	0.01 *	1.00
	AsI MM	−1.04 ± 19.27	−0.19 ± 20.62	−2.09 ± 16.27	1.00	1.00	1.00
	AcI R	−7.93 ± 27.87	−15.69 ± 32.81	5.70 ± 23.47	0.84	0.02 *	0.00 *
	AcI L	−21.20 ± 28.13	−16.77 ± 33.07	3.43 ± 23.47	0.97	0.00 *	0.00 *
	AcI	−15.51 ± 25.30	−16.49 ± 31.48	4.90 ± 22.42	1.00	0.00 *	0.00 *
	TC (%)	−90.43 + 191.56	0.28 ± 2.28	−3.67 ± 76.13	0.02 *	0.03 *	1.00

* Significant differences (*p* < 0.05) between groups (Kruskal-Wallis test).

**Table 4:** Comparison of the mean values (± SD) of bioelectric activity of temporalis anterior (TA), masseter muscle (MM) and electromyographic indices between groups during clenching.

		MTrPs Group	TMDs Group	Control Group	*p* Value MTrPs vs. TMDs	*p* Value MTrPs vs. Controls	*p* Value TMDs vs. Controls
Clenching	TA (µV)	110.22 ± 63.79	89.56 ± 52.39	118.37 ± 63.77	0.29	0.99	0.03 *
	MM (µV)	120.43 ± 91.15	68.30 ± 46.58	133.63 ± 86.03	0.00 *	0.49	0.00 *
	MVC TA (%)	91.82 ± 38.94	116.98 ± 77.34	103.09 ± 42.94	0.02 *	0.32	0.69
	MCV MM (%)	68.95 ± 31.17	77.34 ± 39,59	76.29 ± 32.37	0.67	0.56	1.00
	POC (%)	96.79 ± 33.09	105.51 ± 43.01	97.89 ± 31.02	0.51	1.00	1.00
	AsI TA	−11.47 ± 63.56	−1.22 ± 48.56	−4.47 ± 15.65	0.37	0.07	1.00
	AsI MM	1.44 ± 5.44	1.27 ± 25.64	−1.31 ± 21.33	1.00	1.00	1.00
	AcI R	0.72 ± 28.91	−14.46 ± 26.65	4.12 ± 27.40	0.03 *	1.00	0.00 *
	AcI L	−5.54 ± 28.67	−15.69 ± 34,62	1.09 ± 29.48	0.15	0.61	0.01 *
	AcI	20.42 ± 100.33	−42.52 ± 74.05	3.07 ± 26.23	0.01 *	1.00	0.00 *
	TC (%)	−18.40 ± 68.96	−344.47 ± 4458.64	−14.41 ± 65.91	1.00	1.00	1.00

* Significant differences (*p* < 0.05) between groups (Kruskal-Wallis test).

**Table 5:** Comparison of the mean values (± SD) of bioelectric activity of temporalis anterior (TA), masseter muscle (MM) and electromyographic indices between groups during maximum mouth opening.

		MTrPs Group	TMDs Group	Control Group	*p* Value MTrPs vs. TMDs	*p* Value MTrPs vs. Controls	*p* Value TMDs vs. Controls
Maximum mouth opening	TA (µV)	10.54 ± 7.25	9.71 ± 7.89	8.41 ± 6.63	0.48	0.21	1.00
	MM (µV)	16.45 ± 15.01	10.73 ± 13.37	11.94 ± 14.28	0.01 *	0.33	0.31
	POC (%)	103.49 ± 33.75	135.39 ± 108.85	104.08 ± 32.01	0.17	1.00	0.34
	AsI TA	−2.05 ± 20.10	−0.82 ± 24.49	−0.38 ± 18.97	1.00	1.00	1.00
	AsI MM	0.67 ± 19.50	11.12 ± 28.02	1.27 ± 19.56	0.04 *	1.00	0.12
	AcI R	14.35 ± 24.11	−0.23 ± 36.07	9.60 ± 23.68	0.03 *	1.00	0.28
	AcI L	11.32 ± 26.61	−11.06 ± 31.34	7.65 ± 28.98	0.00 *	1.00	0.02 *
	AcI	13.04 ± 22.63	−3.89 ± 32.12	8.90 ± 24.46	0.01 *	1.00	0.08
	TC (%)	145.17 ± 1141.30	422.55 ± 2474.46	116.17 ± 860.95	0.53	1.00	0.63

* Significant differences (*p* < 0.05) between groups (Kruskal–Wallis test).

## Data Availability

The data presented in this study are available on request from the corresponding author.

## References

[1] Bron C., Dommerholt J.D. (2012). Etiology of Myofascial Trigger Points. Curr. Pain Headache Rep..

[2] Simons D.G., Travel J.G., Simons L.S., Cummings B.D. (1998). Myofascial Pain and Dysfunction: The Trigger Point Manual.

[3] Gerwin R. (2010). Myofascial Pain Syndrome: Here We Are, Where Must We Go?. J. Musculoskelet. Pain.

[4] Zhuang X., Tan S., Huang Q. (2014). Understanding of Myofascial Trigger Points. Chin. Med. J..

[5] Simons D.G. (2004). Review of Enigmatic MTrPs as a Common Cause of Enigmatic Musculoskeletal Pain and Dysfunction. J. Electromyogr. Kinesiol..

[6] Pal U.S., Kumar L., Mehta G., Singh N., Singh G., Singh M., Yadav H.K. (2014). Trends in Management of Myofacial Pain. Natl. J. Maxillofac. Surg..

[7] Shah J.P., Thaker N., Heimur J., Aredo J.V., Sikdar S., Gerber L. (2015). Myofascial Trigger Points Then and Now: A Historical and Scientific Perspective. PM&R.

[8] Pinho-Ribeiro F.A., Verri W.A., Chiu I.M. (2017). Nociceptor Sensory Neuron-Immune Interactions in Pain and Inflammation. Trends Immunol..

[9] Bourgaize S., Newton G., Kumbhare D., Srbely J. (2018). A Comparison of the Clinical Manifestation and Pathophysiology of Myofascial Pain Syndrome and Fibromyalgia: Implications for Differential Diagnosis and Management. J. Can. Chiropr. Assoc..

[10] Runci Anastasi M., Centofanti A., Arco A., Vermiglio G., Nicita F., Santoro G., Cascone P., Anastasi G.P., Rizzo G., Cutroneo G. (2020). Histological and Immunofluorescence Study of Discal Ligaments in Human Temporomandibular Joint. J. Funct. Morphol. Kinesiol..

[11] Kubein-Meesenburg D., Fanghänel J., Ihlow D., Lotzmann U., Hahn W., Thieme K.M., Proff P., Gedrange T., Nägerl H. (2007). Functional State of the Mandible and Rolling-Gliding Characteristics in the TMJ. Ann. Anat..

[12] Cuccia A.M., Caradonna C., Caradonna D. (2011). Manual Therapy of the Mandibular Accessory Ligaments for the Management of Temporomandibular Joint Disorders. J. Am. Osteopath. Assoc..

[13] Durham J., Wassell R. (2011). Recent Advancements in Temporomandibular Disorders (TMDs). Rev. Pain.

[14] Poluha R.L., Grossmann E., Iwaki L.C.V., Uchimura T.T., Santana R.G., Iwaki L. (2018). Myofascial Trigger Points in Patients with Temporomandibular Joint Disc Displacement with Reduction: A Cross-Sectional Study. J. Appl. Oral Sci..

[15] Rener-Sitar K., Celebić A., Mehulić K., Petricević N. (2013). Factors Related to Oral Health Related Quality of Life in TMD Patients. Coll. Antropol..

[16] Ey-Chmielewska H., Teul I., LorkowskI J. (2014). Functional disorders of the temporomandibular joints as a factor responsible for sleep apnoea. Ann. Acad. Med. Stetin..

[17] Facial Pain | National Institute of Dental and Craniofacial Research. https://www.nidcr.nih.gov/research/data-statistics/facial-pain.

[18] Ohrbach R., Dworkin S.F. (1998). Five-Year Outcomes in TMD: Relationship of Changes in Pain to Changes in Physical and Psychological Variables. Pain.

[19] Fernández-de-Las-Peñas C., Galán-Del-Río F., Alonso-Blanco C., Jiménez-García R., Arendt-Nielsen L., Svensson P. (2010). Referred Pain from Muscle Trigger Points in the Masticatory and Neck-Shoulder Musculature in Women with Temporomandibular Disoders. J. Pain.

[20] Alonso-Blanco C., de-la-Llave-Rincón A.I., Fernández-de-las-Peñas C. (2012). Muscle Trigger Point Therapy in Tension-Type Headache. Expert Rev. Neurother..

[21] Olivo S.A., Bravo J., Magee D.J., Thie N.M.R., Major P.W., Flores-Mir C. (2006). The Association between Head and Cervical Posture and Temporomandibular Disorders: A Systematic Review. J. Orofac. Pain.

[22] Alonso-Blanco C., Fernández-de-las-Peñas C., de-la-Llave-Rincón A.I., Zarco-Moreno P., Galán-del-Río F., Svensson P. (2012). Characteristics of Referred Muscle Pain to the Head from Active Trigger Points in Women with Myofascial Temporomandibular Pain and Fibromyalgia Syndrome. J. Headache Pain.

[23] Osiewicz M.A., Lobbezoo F., Loster B.W., Wilkosz M., Naeije M., Ohrbach R. (2013). Research Diagnostic Criteria for Temporomandibular Disorders (RDC/TMD): The Polish Version of a Dual-Axis System for the Diagnosis of TMD.* RDC/TMD Form. Open J. Stomatol..

[24] Peck C.C., Goulet J.-P., Lobbezoo F., Schiffman E.L., Alstergren P., Anderson G.C., de Leeuw R., Jensen R., Michelotti A., Ohrbach R. (2014). Expanding the Taxonomy of the Diagnostic Criteria for Temporomandibular Disorders. J. Oral Rehabil..

[25] Hermens H.J., Freriks B., Disselhorst-Klug C., Rau G. (2000). Development of Recommendations for SEMG Sensors and Sensor Placement Procedures. J. Electromyogr. Kinesiol..

[26] Ferrario V.F., Sforza C., Miani A., D’Addona A., Barbini E. (1993). Electromyographic Activity of Human Masticatory Muscles in Normal Young People. Statistical Evaluation of Reference Values for Clinical Applications. J. Oral Rehabil..

[27] Wieczorek A., Loster J., Loster B.W. (2012). Relationship between Occlusal Force Distribution and the Activity of Masseter and Anterior Temporalis Muscles in Asymptomatic Young Adults. Biomed Res. Int..

[28] De Felício C.M., Sidequersky F.V., Tartaglia G.M., Sforza C. (2009). Electromyographic Standardized Indices in Healthy Brazilian Young Adults and Data Reproducibility. J. Oral Rehabil..

[29] Vozzi F., Favero L., Peretta R., Guarda-Nardini L., Cocilovo F., Manfredini D. (2018). Indexes of Jaw Muscle Function in Asymptomatic Individuals with Different Occlusal Features. Clin. Exp. Dent. Res..

[30] Wieczorek A., Loster J.E. (2015). Activity of the Masticatory Muscles and Occlusal Contacts in Young Adults with and without Orthodontic Treatment. BMC Oral Health.

[31] Ferrario V.F., Tartaglia G.M., Galletta A., Grassi G.P., Sforza C. (2006). The Influence of Occlusion on Jaw and Neck Muscle Activity: A Surface EMG Study in Healthy Young Adults. J. Oral. Rehabil..

[32] Von Elm E., Altman D.G., Egger M., Pocock S.J., Gøtzsche P.C., Vandenbroucke J.P. (2008). STROBE Initiative The Strengthening the Reporting of Observational Studies in Epidemiology (STROBE) Statement: Guidelines for Reporting Observational Studies. J. Clin. Epidemiol..

[33] Peck C., Murray G., Gerzina T. (2008). How Does Pain Affect Jaw Muscle Activity? The Integrated Pain Adaptation Model. Aust. Dent. J..

[34] Ginszt M., Zieliński G., Berger M., Szkutnik J., Bakalczuk M., Majcher P. (2020). Acute Effect of the Compression Technique on the Electromyographic Activity of the Masticatory Muscles and Mouth Opening in Subjects with Active Myofascial Trigger Points. Appl. Sci..

[35] Pietropaoli D., Ortu E., Giannoni M., Cattaneo R., Mummolo A., Monaco A. (2019). Alterations in Surface Electromyography Are Associated with Subjective Masticatory Muscle Pain. Pain Res. Manag..

[36] Manfredini D., Cocilovo F., Favero L., Ferronato G., Tonello S., Guarda-Nardini L. (2011). Surface Electromyography of Jaw Muscles and Kinesiographic Recordings: Diagnostic Accuracy for Myofascial Pain. J. Oral Rehabil..

[37] Mapelli A., Zanandréa Machado B.C., Giglio L.D., Sforza C., De Felício C.M. (2016). Reorganization of Muscle Activity in Patients with Chronic Temporomandibular Disorders. Arch. Oral Biol..

[38] Simons D.G., Travell J.G., Simons L.S. (1999). Travell & Simons’ Myofascial Pain and Dysfunction: The Trigger Point Manual.

[39] Couppé C., Torelli P., Fuglsang-Frederiksen A., Andersen K.V., Jensen R. (2007). Myofascial Trigger Points Are Very Prevalent in Patients with Chronic Tension-Type Headache: A Double-Blinded Controlled Study. Clin. J. Pain.

[40] Alonso-Blanco C., Fernández-de-las-Peñas C., Fernández-Mayoralas D.M., de-la-Llave-Rincón A.I., Pareja J.A., Svensson P. (2011). Prevalence and Anatomical Localization of Muscle Referred Pain from Active Trigger Points in Head and Neck Musculature in Adults and Children with Chronic Tension-Type Headache. Pain Med..

[41] Fernández-de-las-Peñas C., Fernández-Mayoralas D.M., Ortega-Santiago R., Ambite-Quesada S., Palacios-Ceña D., Pareja J.A. (2011). Referred Pain from Myofascial Trigger Points in Head and Neck-Shoulder Muscles Reproduces Head Pain Features in Children with Chronic Tension Type Headache. J. Headache Pain.

[42] Fernández-de-Las-Peñas C., Cuadrado M.L., Pareja J.A. (2007). Myofascial Trigger Points, Neck Mobility, and Forward Head Posture in Episodic Tension-Type Headache. Headache.

[43] Fernández-de-Las-Peñas C., Ge H.-Y., Arendt-Nielsen L., Cuadrado M.L., Pareja J.A. (2007). Referred Pain from Trapezius Muscle Trigger Points Shares Similar Characteristics with Chronic Tension Type Headache. Eur. J. Pain.

[44] La Touche R., Fernández-de-Las-Peñas C., Fernández-Carnero J., Díaz-Parreño S., Paris-Alemany A., Arendt-Nielsen L. (2010). Bilateral Mechanical-Pain Sensitivity over the Trigeminal Region in Patients with Chronic Mechanical Neck Pain. J. Pain.

[45] De-la-Llave-Rincon A.I., Alonso-Blanco C., Gil-Crujera A., Ambite-Quesada S., Svensson P., Fernández-de-Las-Peñas C. (2012). Myofascial Trigger Points in the Masticatory Muscles in Patients with and without Chronic Mechanical Neck Pain. J. Manip. Physiol. Ther..

[46] Testa M., Geri T., Gizzi L., Falla D. (2017). High-Density EMG Reveals Novel Evidence of Altered Masseter Muscle Activity During Symmetrical and Asymmetrical Bilateral Jaw Clenching Tasks in People With Chronic Nonspecific Neck Pain. Clin. J. Pain.

[47] Ohlmann B., Waldecker M., Leckel M., Bömicke W., Behnisch R., Rammelsberg P., Schmitter M. (2020). Correlations between Sleep Bruxism and Temporomandibular Disorders. J. Clin. Med..

[48] Pihut M., Górnicki M., Orczykowska M., Zarzecka E., Ryniewicz W., Gala A. (2020). The Application of Radiofrequency Waves in Supportive Treatment of Temporomandibular Disorders. Pain Res. Manag..

[49] Eraslan R., Kılıç K., Etöz M., Soydan D. (2020). The Evaluation of Agreement between High-Frequency Ultrasonography and Research Diagnostic Criteria for the Diagnosis of Temporomandibular Joint Internal Derangements. J. Indian Prosthodont. Soc..

[50] Rehm D.D., Progiante P.S., Pattussi M.P., Pellizzer E.P., Grossi P.K., Grossi M.L. (2020). Sleep Disorders in Patients with Temporomandibular Disorders (TMD) in an Adult Population-Based Cross-Sectional Survey in Southern Brazil. Int. J. Prosthodont..

